# Muscle and serum myostatin expression in type 1 diabetes

**DOI:** 10.14814/phy2.14500

**Published:** 2020-07-11

**Authors:** Athan G. Dial, Cynthia M. F. Monaco, Grace K. Grafham, Nadya Romanova, Jeremy A. Simpson, Mark A. Tarnopolsky, Christopher G. R. Perry, Evangelia Kalaitzoglou, Thomas J. Hawke

**Affiliations:** ^1^ Department of Pathology and Molecular Medicine McMaster University Hamilton ON Canada; ^2^ Department of Human Health and Nutritional Sciences University of Guelph Guelph ON Canada; ^3^ Deparment of Pediatrics McMaster University Hamilton ON Canada; ^4^ School of Kinesiology and Health Science York University Toronto ON Canada; ^5^ Barnstable Brown Diabetes Center and Department of Pediatrics University of Kentucky Lexington KY USA

**Keywords:** GDF‐8, myostatin, skeletal muscle, T1D, TGF‐family members

## Abstract

Type 1 diabetes (T1D) has been reported to negatively affect the health of skeletal muscle, though the underlying mechanisms are unknown. Myostatin, a myokine whose increased expression is associated with muscle‐wasting diseases, has not been reported in humans with T1D but has been demonstrated to be elevated in preclinical diabetes models. Thus, the purpose of this study was to determine if there is an elevated expression of myostatin in the serum and skeletal muscle of persons with T1D compared to controls. Secondarily, we aimed to explore relationships between myostatin expression and clinically important metrics (e.g., HbA_1c_, strength, lean mass) in women and men with (*N* = 31)/without T1D (*N* = 24) between 18 and 72 years old. Body composition, baseline strength, blood sample and vastus lateralis muscle biopsy were evaluated. Serum, but not muscle, myostatin expression was significantly elevated in those with T1D versus controls, and to a greater degree in T1D women than T1D men. Serum myostatin levels were not significantly associated with HbA_1c_ nor disease duration. A significant correlation between serum myostatin expression and maximal voluntary contraction (MVC) and body fat mass was demonstrated in control subjects, but these correlations did not reach significance in those with T1D (MVC: *R* = 0.64 controls vs. *R* = 0.37 T1D; Body fat: *R* = −0.52 controls/*R* = −0.02 T1D). Collectively, serum myostatin was correlated with lean mass (*R* = 0.45), and while this trend was noted in both groups separately, neither reached statistical significance (*R* = 0.47 controls/*R* = 0.33 T1D). Overall, while those with T1D exhibited elevated serum myostatin levels (particularly females) myostatin expression was not correlated with clinically relevant metrics despite some of these relationships existing in controls (e.g., lean/fat mass). Future studies will be needed to fully understand the mechanisms underlying increased myostatin in T1D, with relationships to insulin dosing being particularly important to elucidate.

## Graphical abstract

Myostatin, a negative regulator of muscle size/health is increased in type 1 diabetes. Relative increases in females are greater than in males with the disease.

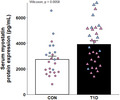

## 1 INTRODUCTION

Skeletal muscle, by virtue of its mass, is the largest metabolic organ in the body. Nearly 80% of insulin‐stimulated glucose uptake after a meal occurs in muscle (DeFronzo, 2009; Honka et al., 2018). As such, alterations to the health of skeletal muscle would have profound effects on glycemic control. Despite the major role of skeletal muscle in both our physical capacity for exercise and activities of daily living, our knowledge of changes to muscle health during type 1 diabetes (T1D) is currently limited. T1D has been associated with decreased muscle mass and strength (Maratova et al., 2018; Mori et al., 2017; Orlando, Balducci, Bazzucchi, Pugliese, & Sacchetti, 2017), as well as impaired muscle ultrastructure and energy production even in the absence of other co‐morbidities (Cree‐Green et al., 2015; Monaco et al., 2019). However, the underlying mechanism(s) behind these alterations to skeletal muscle health remain to be elucidated.

Myostatin is a potent negative regulator of muscle growth. Primarily synthesized in skeletal muscle, myostatin binds to its receptor complex activin IIB on the cell surface resulting in a cascade of signaling from the cell surface to the nucleus (Elliott et al., 2012). Ultimately, myostatin targets a number of transcription factors that promote muscle atrophy (Elkina, von Haehling, Anker, & Springer, 2011). Elevations in myostatin expression have been observed repeatedly in humans with muscle wasting conditions (Elliott et al., 2012) and in metabolic disorders like type 2 diabetes (Brandt et al., 2012). Evidence in rodent models suggests that myostatin expression is elevated in type 1 diabetes (Chen, Cao, Ye, & Zhu, 2009) and elevated myostatin expression is associated with impaired insulin signaling/sensitivity (Brandt et al., 2012; Chen et al., 2009; Wang et al., 2012).

Despite accumulating evidence for a role of myostatin in the maintenance of skeletal muscle function and metabolic health, its expression in the blood and muscle of those with type 1 diabetes has not previously been reported. Thus, the purpose of the current study was to investigate the expression of myostatin in men and women with type 1 diabetes and, secondarily, explore relationships between myostatin expression and clinically important health metrics.

## 2 MATERIALS AND METHODS

### 2.1 Participants

Adult men/women with type 1 diabetes (*n* = 31; 21 female, 10 male) and matched control participants without diabetes (*n* = 24; 15 female, 9 male) 18–72 years of age were recruited for this study. Participants were matched for age, sex, and self‐reported physical activity levels, and were told to refrain from exercise 24 hr preceding either study visit. Detailed subject characteristics and exclusion criteria are available in Table 1.

**TABLE 1:** Subject characteristics. Adult men/women with type 1 diabetes and matched control participants without diabetes were recruited for this study. Participants were not eligible to participate in the study if they matched any one of the following criteria: smoke or use any tobacco products, prediabetes, type 2 diabetes, statin‐induced myopathy or experience myalgia, use assistive walking devices, chronically medicate with any analgesic or anti‐inflammatory drug(s), corticosteroids or non‐steroidal anti‐inflammatories or prescription strength acne medications, use medications known to affect muscle metabolism, heart disease, rheumatoid arthritis, poor lung function, uncontrolled hypertension, stage 3 neuropathy, severe retinopathy, or any health conditions that put the subject at risk during this study

Characteristic	CON		T1DM	
	Female	Male	Female	Male
*n*	15	9	21	10
Age (years)	32‐ ± 3.2	27‐ ± 2.5	32‐ ± 2.4	32‐ ± 4.9
Height (m)	1.64 ± 0.02	1.77 ± 0.01^†^	1.64 ± 0.01	1.77 ± 0.02^†^
Weight (kg)	62.55 ± 2.95	77.45 ± 2.01^†^	70.45 ± 3.48	84.06 ± 3.94^†^
BMI (kg/m^2^)	23.2 ± 0.79	24.64 ± 0.68	26.04 ± 1.17*	26.66 ± 1.04
HbA1c (%)	5.21 ± 0.08	5.42 ± 0.16	7.94 ± 0.31*	7.18 ± 0.28*
T1DM Duration (years)	—	—	16.13 ± 2.49	21.18 ± 4.71
Diabetes Onset (years of age)	—	—	16.52 ± 2.15	11.00 ± 1.94

Data are expressed as mean ± *SEM*.

* Significantly different versus same‐sex control, *p* < .05.

† Significantly different versus female of same group, *p* < .05.

### 2.2 Ethics

Prior to giving written informed consent, all participants were given oral and written information about the experimental procedures. All procedures herein were approved by the Research Ethics Board at York University (REB #e2013‐032) and Hamilton Integrated Research Ethics Board (REB #5344 and #5355) and conformed to all declarations on the use of human participants as research participants.

### 2.3 Study protocol

Participants visited the laboratory for baseline strength measurement procedure (maximal voluntary contractions, MVCs) using a Biodex dynamometer (Biodex System 3; Biodex Medical Systems) and a subset of participants (Controls: 11 female, 6 male; T1D: 12 female, 5 male) underwent body composition assessment via dual‐energy X‐ray absorptiometry (DEXA; GE Healthcare). Participants were instructed to refrain from caffeine and alcohol 24 hr prior to visiting the laboratory and were instructed to consume a standardized meal 1.5 to 2 hr prior to their visit. Participants with T1D were also instructed to continue their habitual use of insulin.

### 2.4 Muscle and blood measurements

Participants underwent a biopsy of the vastus lateralis muscle and a venous blood sample. Biopsies were obtained from the mid‐portion of the vastus lateralis under local anesthetic (1% lidocaine) using manual suction. A resting blood sample was obtained from the antecubital vein immediately following the muscle biopsy. HbA_1c_ levels were determined by the local core lab facility at McMaster University.

### 2.5 Myostatin measurements

Serum myostatin was measured using a commercially available ELISA kit (R&D Systems, DGDF80). Muscle myostatin was measured by Western blot from skeletal muscle lysates using a custom‐derived myostatin antibody (gifted by Dr. Jeremy Simpson), and a 15 kDa band representing myostatin in its monomeric form was quantified, as described previously (Dasarathy, Dodig, Muc, Kalhan, & McCullough, 2004). More detailed information including a representative blot with control is shown in ESM Figure 1.

**FIGURE 1:**
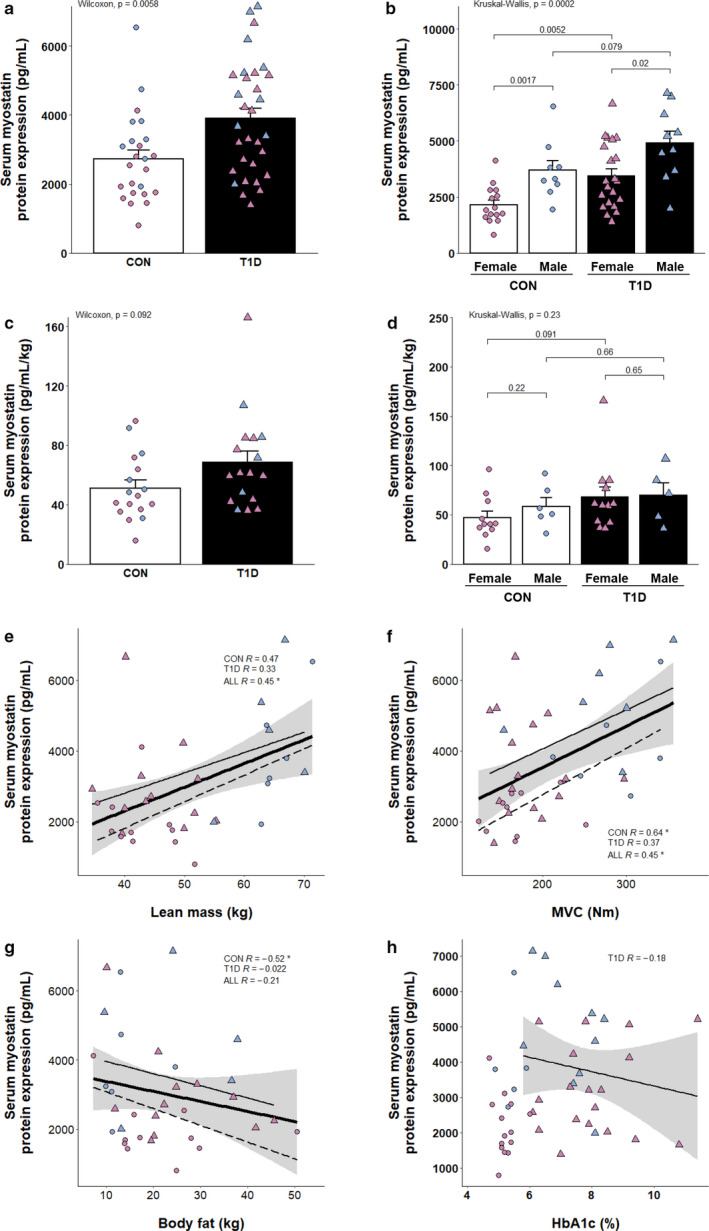
Elevated expression of serum myostatin and its correlates in T1D. (a) Serum myostatin expression as measured by ELISA (CON, *n* = 24; T1D, *n* = 31). (b) Serum myostatin expression as measured by ELISA, men and women analyzed separately (CON, *n* = 24; T1D, *n* = 31). (c) Serum myostatin expression as measured by ELISA normalized to lean mass (CON, *n* = 17; T1D, *n* = 17). (d) Serum myostatin expression as measured by ELISA normalized to lean mass, men and women analyzed separately (CON, *n* = 17; T1D, *n* = 17). Scatter plots summarizing the relationship between myostatin and various metrics are shown in (e–h). (e) Serum myostatin versus lean mass in kilograms (CON, *n* = 17; T1D, *n* = 17). (f) Serum myostatin versus maximal voluntary contraction strength (MVC; CON, *n* = 16; T1D, *n* = 23). (g) Serum myostatin versus whole‐body fat in kilograms (CON, *n* = 17; T1D, *n* = 17). (h) Serum myostatin versus HbA_1c_ (T1D, *n* = 29). For individual data points, circles indicate control participants, triangles indicate T1D, blue fill indicates men, purple fill indicates women. For regression lines, dashed lines indicate control participants, solid thin lines indicate T1D, solid thick lines indicate overall regression line, gray shading indicates standard error. Spearman's rho (displayed as *R*) and *p*‐values are displayed within graphical summary. *p < .05

### 2.6 Microarray data mining and analysis

Microarray datasets were accessed through the Gene Expression Omnibus (GEO) online database according to the following accession codes and were given shorthand names based on the author of original publications: GSE22309 (Wu), GSE18732 (Gallagher), GSE121 (Yang), GSE19420 (vanTienen), GSE25462 (Patti). GSE121 was preprocessed as described in its source publication. All other datasets were preprocessed using Affy package in an R environment with RMA background correction, quantile normalization and log_2_ transformation (Gautier, Cope, Bolstad, & Irizarry, 2004). GSE25462 contains multiple experimental groups of which only two were used for contrasts—Type 2 Diabetes participants (T2D) participants versus control participants with no family history of T2D. For each dataset, gene symbols were obtained using the AnnotationDBI package in R. Differential expression analysis was performed using limma package in R (Ritchie et al., 2015).

### 2.7 Statistical analysis

Bar graphs are presented as mean ± *SEM* with data points overlaid. Data points are presented as circles (controls) and triangles (T1D) with purple (female) and blue (male) colors to define sex. Participant characteristics are expressed as mean ± *SEM*. Not all samples were available for all analyses and the specific number of participants for each group are highlighted in the overlain dot plots and expressly written out in the figure legend. Due to multiple non‐normally distributed variables, nonparametric statistics were utilized in this study. Wilcoxon–Mann–Whitney tests were performed to compare myostatin measurements between groups (i.e., CON and T1D). Kruskal–Wallis was used for comparisons of multiple groups (i.e., group and sex), followed by post hoc Wilcoxon–Mann–Whitney tests with Bonferroni–Holm correction for multiple comparisons. Correlational analyses were performed with Spearman's rho (herein denoted as *R*). Statistical significance was established at *p* <0 .05. All analyses were carried out in the R environment for statistical computing (R Foundation for Statistical Computing).

## 3 RESULTS

### 3.1 Circulating myostatin expression is elevated in adults with type 1 diabetes

Serum myostatin was significantly elevated in adults with type 1 diabetes compared to matched persons without diabetes (Figure 1a). When separated by sex, women had 30%–40% less myostatin in circulation than men, independent of disease (Figure 1b), consistent with previous observations (Nishikawa et al., 2017). In women with type 1 diabetes, serum myostatin was significantly higher relative to control women. There was also a notably elevated serum myostatin expression in men with type 1 diabetes versus controls (~33%) though this difference did not meet statistical significance (*p* = 0.08). We then investigated the ratio of serum myostatin to lean mass to determine if the absolute muscle mass of participants influenced circulating myostatin concentrations and could be contributing to the sex differences observed. Interestingly, when normalized to muscle mass, expression remained 33% higher in those with type 1 diabetes (*p* = 0.09; Figure 1c). When separated by sex, a ~20%–40% elevated expression was observed between T1D and the same‐sex control counterparts, but these differences were not statistically significant (*p* = 0.09 females, *p*  =0 .66 males; Figure 1d).

### 3.2 Elevated serum myostatin levels are associated with increased lean mass, and maximal strength

When all participants were combined, serum myostatin showed a significant positive correlation with lean mass and MVC (Figure 1e,f). There were no observable differences in MVC between groups (*p* = 0.54; ESM Figure 2), yet, when separated by group, controls displayed a significant positive correlation with MVC while the weaker association in T1D participants fell short of the threshold for significance (Figure 1f). In line with this, serum myostatin was significantly, and negatively, associated with body fat mass, again only in control participants (*R* = −0.52; *p* = 0.03; Figure 1g). Interestingly, we observed that circulating myostatin expression was not associated with HbA_1c_ (*R* = −0.18; Figure 1h) nor duration of disease in those with T1D (*R* = 0.04; ESM Figure 3).

**FIGURE 2:**
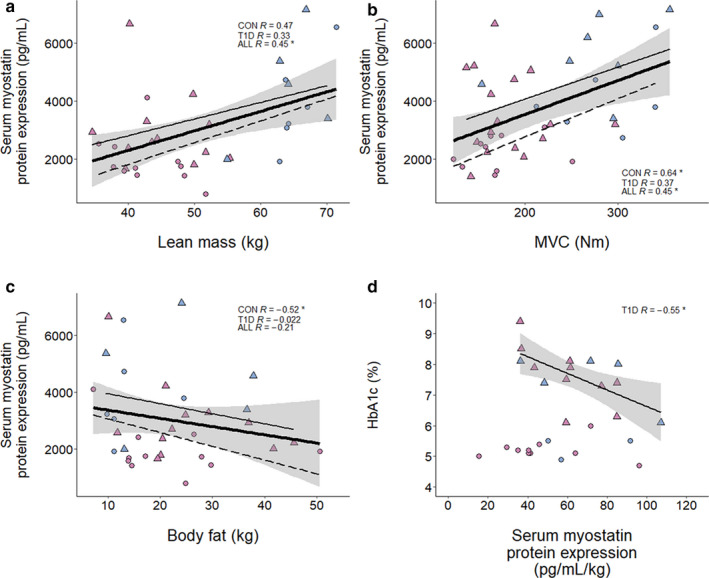
Correlates of muscle myostatin protein expression with and without T1D. (a) Skeletal muscle myostatin expression as measured by Western blot (CON, *n* = 20; T1D, *n* = 20), with inlaid representative blot displaying 15 kDa band representing monomeric myostatin, and Coomassie Blue loading control. (b) Skeletal muscle myostatin expression, men and women analyzed separately (CON, *n* = 20; T1D, *n* = 20). Scatter plots summarizing the relationship between myostatin and various metrics are shown in (c–f). (c) Skeletal muscle myostatin versus lean mass in kilograms (CON, *n* = 14; T1D, *n* = 12). (d) Skeletal muscle myostatin versus MVC (CON, *n* = 10; T1D, *n* = 10). (e) Skeletal muscle myostatin versus body fat in kilograms (CON, *n* = 14; T1D, *n* = 12). (f) Skeletal muscle myostatin versus HbA_1c_ in percent (T1D, *n* = 19). Analysis of publicly available microarray datasets are shown in (h, i). (h) Bar plot of muscle myostatin mRNA log_2_ fold change values relative to healthy control group from each respective study. White bars indicate insulin‐resistant (IR) groups, while black bars indicate T2D groups. *p*‐values are displayed outside of each bar. (i) Scatter plot depicting the correlation between muscle myostatin log_2_ mRNA expression and HbA_1c_ in percent. Data obtained from GSE18732. For individual data points, circles indicate control participants, triangles indicate T1D, blue fill indicates men, purple fill indicates women. For regression lines, dashed lines indicate control participants, solid thin lines indicate T1D, solid thick lines indicate overall regression line, gray shading indicates standard error. Spearman's rho (displayed as *R*) and *p*‐values are displayed within graphical summary. *p < .05

**FIGURE 3:**
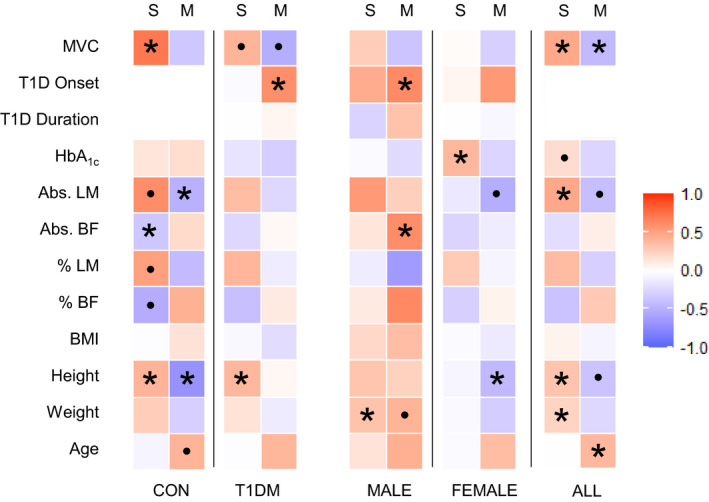
Summary of correlative analyses in groupings of the present study. Heat map summarizing strength of correlation between clinical metrics in this study and myostatin protein expression in the serum (S, top) and muscle (M, top). Variables are displayed along the left side including: maximal voluntary contraction (MVC), age of T1D diagnosis (T1D Onset), duration of T1D (T1D Duration), HbA_1c_, absolute lean body mass (Abs. LM), absolute body fat (Abs. BF), relative lean body mass (% LM), relative body fat (% BF), body mass index (BMI), height, weight, and age. Participants have been grouped as healthy controls (CON), diabetic participants (T1D), males (MALE), females (FEMALE), and overall cohort (ALL). For each square, red fill represents positively correlated variables, blue fill represents negative correlation. Intensity of fill represents the strength of correlation. *p < 0.05; ●*p* < 0.1

### 3.3 T1D and non‐diabetic muscle express comparable myostatin protein

In contrast to circulating myostatin, skeletal muscle myostatin levels were not differentially affected by disease or sex (Figure 2a,b). Muscle myostatin was significantly and negatively associated with lean mass in control participants (*R* = −0.6; *p* = 0.03; Figure 2c) but not in T1D (*p* = −0.17; Figure 2c). In line with this, elevated myostatin expression was also associated with decreased MVC in all participants (*R* = −0.46; *p* = 0.04; Figure 2d). Contrary to serum myostatin expression, muscle myostatin levels were not significantly associated with body fat or HbA_1c_ (Figure 2e,f).

### 3.4 Myostatin expression in diabetic muscle – context from microarray studies

The question arose of whether there was a relationship between insulin sensitivity and myostatin expression, in part because the current data differed from previous studies in T2D where muscle myostatin mRNA and/or protein is elevated in the diabetic state (Brandt et al., 2012; Hittel, Berggren, Shearer, Boyle, & Houmard, 2009). Unfortunately, we did not have direct results of insulin sensitivity in the present study. However, to further contextualize our results to the broader literature, we surveyed GEO for microarray experiments involving skeletal muscle biopsies from diabetic participants. While we were unable to find any such datasets with muscle biopsies from those with T1D, we were able to examine a cohort of microarray datasets involving muscle biopsies from participants with insulin‐resistance (IR) and/or T2D. Interestingly, muscle myostatin mRNA was expressed to similar levels in insulin‐resistant/T2D muscle in 4 out of 5 of the experiments analyzed (Figure 2h). Gallagher and colleagues (Gallagher et al., 2010) have included an excellent set of participant data as part of GSE18732 that allowed for correlational analyses between myostatin mRNA expression and variables related to IR/diabetes and overall metabolic health. The data from GSE18732, as well as the data from the current study suggest there is not a significant association between HbA_1c_ and muscle myostatin in human participants overall (Figure 2f,i), nor when divided into experimental groups (ESM Figure 4). However, myostatin expression from GSE18732 was positively correlated with metrics of insulin resistance such as the Homeostasis Model Assessment (HOMA) 1/2 (Table 2). An expanded table summarizing a selection of correlates of myostatin mRNA expression from this GSE18732 can be found in Table 2.

**TABLE 2:** Clinically relevant correlates of myostatin mRNA expression. A selection of subject characteristics recorded in GSE18732 is shown, summarized by Spearman's rho. Experimental groups analyzed separately (normal glucose tolerance, CON; impaired glucose tolerance IR; type 2 diabetic, T2DM), as well as the entire cohort (ALL)

Variable	ALL	CON	IR	T2DM
Age	−0.17	−0.31*	−0.16	−0.03
BMI	0.29*	0.30*	0.50*	0.12
Glucose, baseline	0.11	0.17	0.13	0.05
HbA1c	0.02	−0.05	0.00	−0.08
HOMA1 IR	0.35*	0.33*	0.71*	0.33*
HOMA2 IR	0.41*	0.33*	0.74*	0.39*
Insulin, baseline	0.39*	0.32*	0.72*	0.34*
Total Fat Mass	0.22*	0.30*	0.38	−0.02
Total Lean Mass	0.25*	0.22	0.49*	0.09
Weight	0.29*	0.32*	0.55*	0.06

* *p* < .05.

A heat map summarizing the correlative data analyzed in the current study can be found in Figure 3.

## 4 DISCUSSION

Here, we report for the first time, the myostatin expression levels in the blood and muscle of persons with type 1 diabetes. Our data reveal that people with T1D exhibited elevated circulating myostatin levels, with the relative increase in females ‐ greater than that in males. In contrast, protein expression of myostatin in the skeletal muscle was similar between cohorts and not different between sexes. Furthermore, we show that glycemic control (as measured by HbA_1c_) was not associated with myostatin expression in the serum, nor the skeletal muscle.

In this study, we observed, collectively, that elevated serum myostatin was associated with leaner and stronger participants (Figure 1e‐g). This is in line with a recent observation in which elevated myostatin levels were observed in elderly men who were more active and less frail (Arrieta et al., 2019), but is contrary to other findings (Chew et al., 2019). Furthermore, a positive correlation between myostatin expression and adiposity has previously been observed in obese participants (Hittel et al., 2009). The reason(s) underlying why conditions such as obesity exhibit an increase in muscle myostatin expression versus the relationship observed here are unknown, but may pertain to the role of myostatin in insulin sensitivity previously reported (Hittel et al., 2009) and supported by some, but not all of our correlative analysis of myostatin expression (such as in GSE18732; Figure 2i; Table 2). Likely, insulin‐resistance is a less severe trait in the current cohort of participants, who were otherwise healthy, active individuals without other co‐morbidities. Interestingly, neither serum nor muscle myostatin expression was related to HbA_1c_ (Figures 1h and 2f,i) lending support for a potential role of myostatin in the development of insulin resistance, as previously suggested (Amor et al., 2018; Brandt et al., 2012; Hittel et al., 2009), that is distinct from its effects on systemic glycemic control as measured by HbA_1c_. Thus, these current protein expression data in skeletal muscle adds an important piece of evidence to the T1D literature. The lack of association between myostatin protein expression and HbA_1c_ in T1D suggests that (a) the involvement of myostatin in the development insulin resistance does not necessarily extend to an association with HbA_1c_, and/or (b) obesogenic factors that are often associated with T2D participants may be a key feature of the pathology that is driving a higher myostatin expression, as suggested by previous work in obese individuals (Amor et al., 2018; Hittel et al., 2009). Indeed, the average BMI of T1D patients in the current study was ~26 as opposed to ~30 in previous work where elevated muscle myostatin was observed in T2D (Brandt et al., 2012).

Taken together, the data suggest that myostatin expression covaries with muscle mass. In fact, in a stable disease condition, such as the current study cohort, it appears that serum myostatin expression reflects the amount of muscle mass producing myostatin in the body. Therefore, differences in muscle mass between cohorts may be important to consider whenever interpreting circulating myostatin expression levels in humans with or without a pathology. For example, situations where atrophy is a prevalent symptom may display higher (Barreiro & Jaitovich, 2018; Man et al., 2010; Plant et al., 2010), lower (Loumaye et al., 2015) or comparable expression of myostatin (Brandt et al., 2012) in circulation. Equivocal findings in this area suggest that serum expression of myostatin may have disease‐specific responses that are secondary to each pathology. Caution is warranted when interpreting circulating expression levels as a singular diagnostic signal of poor muscle health.

Historically, it has often been posited that elevated myostatin expression elicits negative effects on metrics of muscle health and metabolism. However, the analyses here along with the growing number of studies observing myostatin as a biomarker in the context of chronic and metabolic diseases reveals the complex and somewhat unpredictable behavior of the myokine (Arrieta et al., 2019; Brandt et al., 2012; Elliott et al., 2012; Hittel et al., 2009). Our interrogation of available microarray data suggest a relationship between muscle myostatin mRNA and measures of insulin resistance are present in T2D, and though not investigated here, future studies defining the relationship between myostatin expression and the development of insulin resistance in persons with T1D would be of significant interest to help combat the comorbidities associated with insulin resistance in this population.

## 5 CONCLUSIONS

In conclusion, this is the first observation of myostatin expression in persons with T1D and reveals that the myokine is expressed to a higher degree in the serum of persons with T1D, with leaner individuals displaying the highest levels of expression. Furthermore, we also observed a sexual dimorphic response with T1D women exhibiting a larger relative increase in serum myostatin (versus control females) compared to the same comparison in men. Overall, data here and elsewhere (Arrieta et al., 2019) suggest that, in our otherwise healthy cohort of persons with T1D, myostatin may be operating as a homeostatic, rather than pathological, regulator of muscle mass and may be associated with a leaner, more muscular phenotype in these men and women.

## CONFLICT OF INTEREST

The authors declare that there is no duality of interest associated with this manuscript.

## AUTHOR CONTRIBUTION

AGD, CMFM, and TJH designed the experiments. AGD and TJH wrote the manuscript. AGD and CMFM performed the experiments. AGD analyzed the data. AGD, CMFM, EK, and TJH interpreted the data. JAS and NR developed and validated the antibody and assisted in western blotting. MAT and CGRP performed muscle biopsies. AGD, CMFM, and GKG collected and processed muscle samples. All authors edited the manuscript. All authors provided final approval of the version to be published. All people designated as authors qualify for authorship, and all those who qualify for authorship are listed. TJH is the guarantor of this work and, as such, had full access to all the data in the study and take responsibility for the integrity of data and the accuracy of the data analysis.

## Supporting information



Fig S1‐4
