# Supplementary material for: Muscle and serum myostatin expression in type 1 diabetes
Source: Physiol Rep. 2020 Jul 11;8(13):e14500. doi: 10.14814/phy2.14500 (PMC7354085; doi:10.14814/phy2.14500)
Supplement: Supplementary file 1 — Fig S1‐4 [file PHY2-8-e14500-s001.pdf]

Electronic Supplementary Material (ESM)

ESM Table 1. Western blotting reagents used in the present study.
* = Custom derived antibody

Reagent	Name	Host	Dilution	MW (kDa)	Manufacturer	Catalogue Number
**Primary antibody**	Myostatin	Rabbit	1:200	15, 25, 42	Simpson Lab* Guelph, ON, CAN	N/A
**Secondary antibody**	Alkaline Phosphatase AffiniPure Goat Anti-Rabbit IgG (H+L)	Goat	1:2,000	-	Jackson ImmunoResearch Laboratories Inc. West Grove, PA, USA	111-055-003
**Blocking reagent**	1X Blocking Reagent for ELISA	-	1:1	-	Roche Basel, SWI	11112589001

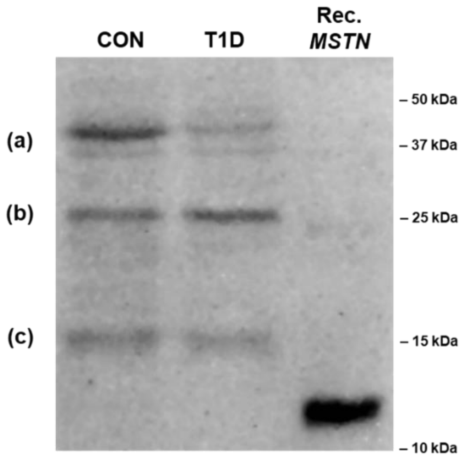

ESM Figure 1. Myostatin western blot with positive control. Representative full-lane western blot, depicting major observable bands at (a) ~42 kDa representing myostatin associated with its propeptide, (b) ~25 kDa representing the dimeric protein, and (c) ~15 kDa representing the unprocessed monomeric form of the protein, as previously observed (Dasarathy et al., 2004). Recombinant myostatin protein used to derive the antibody was utilized as a positive control on all blots (Rec. MSTN). For the present study, we display results for the 15 kDa band, in order to be most representative of the positive control.

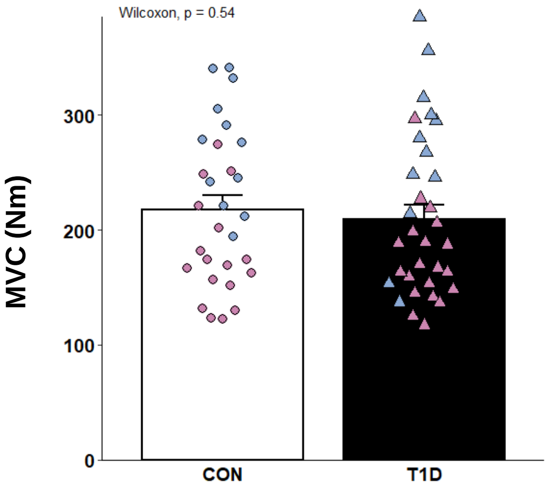

ESM Figure 2. Comparable muscle strength in T1DM and non-diabetic participants. Bar plot displaying maximal voluntary contraction (MVC) strength. For individual data points, circles indicate control participants, triangles indicate T1DM, blue fill indicates men, purple fill indicates women. p-values are displayed within graphical summary.

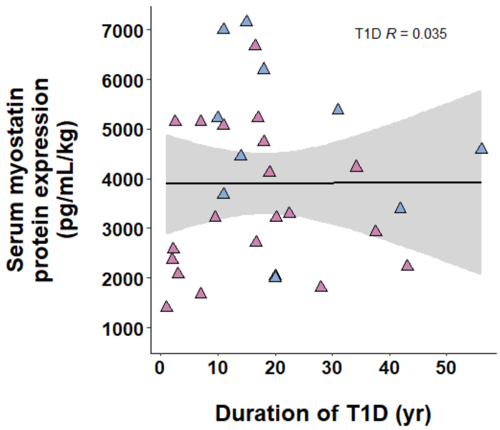

ESM Figure 3. Serum myostatin is not associated with duration of diabetes. For individual data points, circles indicate control participants, triangles indicate T1DM, blue fill indicates men, purple fill indicates women. For regression lines, solid thin lines indicate T1DM, solid thick lines indicate overall regression line, grey shading indicates standard error. Spearman’s rho (displayed as R) is displayed within graphical summary.

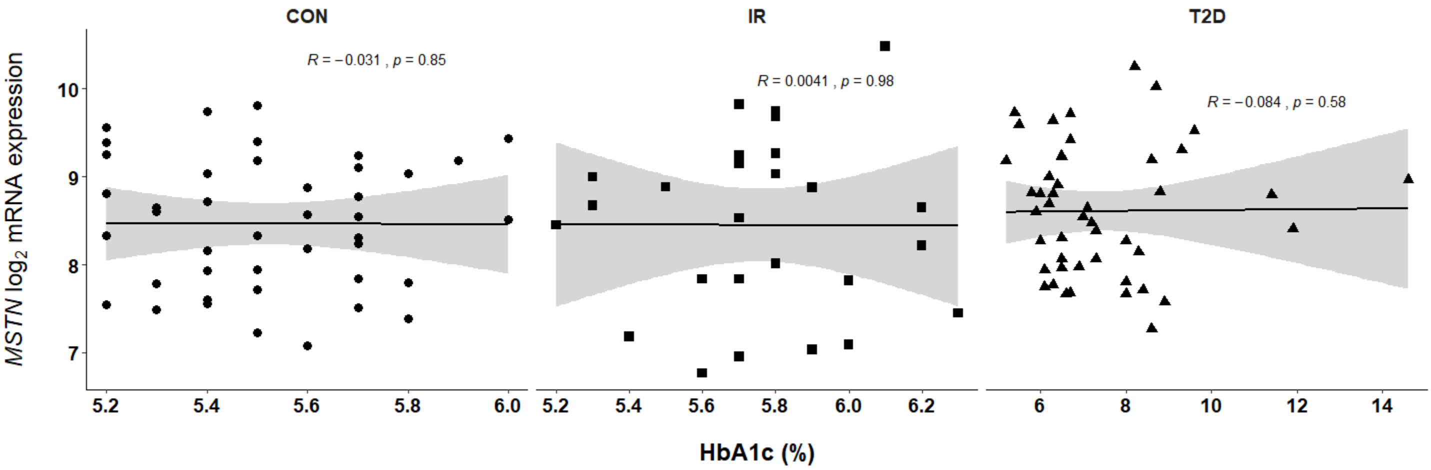

ESM Figure 4. Scatter plots displaying myostatin $\log_2$ mRNA expression vs HbA_{1c} from GSE18732. Experimental groups analysed separately (normal glucose tolerance, CON; impaired glucose tolerance IR; type 2 diabetic, T2DM). Solid black lines indicate regression lines, grey shading indicates standard error. Spearman’s rho (displayed as R) and p-values are displayed within graphical summary.
